# Role of CXCR3 and CXCR6 on Circulating T Cells in Patients With Parkinson's Disease

**DOI:** 10.1111/sji.70090

**Published:** 2026-02-08

**Authors:** Yufeng Zhang, Zhuangzhuang Ren, Shuangshuang Jia, Jinming Han, Xiaoling Zhong, Bo Fu, Haoran Wang, Shiping Xu, Tingting Li, Feng Qiu

**Affiliations:** ^1^ Medical School of Chinese PLA Beijing China; ^2^ Department of Neurology, the First Medical Center Chinese PLA General Hospital Beijing China; ^3^ Department of Neurology, the Eighth Medical Center Chinese PLA General Hospital Beijing China; ^4^ Navy Clinical College, the Fifth School of Clinical Medicine Anhui Medical University Hefei Anhui China; ^5^ State Key Laboratory of Kidney Diseases National Clinical Research Center for Kidney Diseases Beijing China; ^6^ Department of Neurology Huaihe Hospital of Henan University Kaifeng China; ^7^ Department of Neurology, Xuanwu Hospital Capital Medical University Beijing China; ^8^ Department of Nephrology, the First Medical Center Chinese PLA General Hospital Beijing China; ^9^ Department of Gastroenterology, the Second Medical Center Chinese PLA General Hospital Beijing China; ^10^ National Clinical Research Center for Geriatric Diseases Chinese PLA General Hospital Beijing China

**Keywords:** cellular immunity, CXCR3, CXCR6, Parkinson's disease (PD), peripheral inflammation

## Abstract

Immune dysregulation is involved in Parkinson's disease (PD), but the roles of C‐X‐C motif chemokine receptor 3 (CXCR3)/chemokine receptor 6 (CXCR6) on T cells and their correlation with peripheral inflammation remain unclear. This study investigated their expression on peripheral blood T cells and correlation with inflammation in PD. A total of 36 PD patients and 26 healthy controls were enrolled; their clinical information and laboratory test results (including the systemic immunoinflammatory index [SII], neutrophil‐to‐lymphocyte ratio [NLR], monocyte‐to‐lymphocyte ratio [MLR], platelet‐to‐lymphocyte ratio [PLR], monocyte‐to‐high‐density lipoprotein ratio [MHR], and erythrocyte distribution width over platelets ratio [RPR]) were recorded. Meanwhile, a multicolour flow cytometry protocol using six cell surface antibodies (CD3, CD4, CD8, CD45RO, CXCR3, and CXCR6) was applied. The results showed that CD8^+^ T cells were significantly reduced in the PD group compared with healthy controls (*p* < 0.001), and CXCR3 expression was significantly increased on peripheral blood CD4^+^ effector T cells and CD8^+^ T cells of PD patients (*p* < 0.001). Additionally, CXCR6 expression was significantly elevated on cytotoxic T lymphocytes (CTLs) (*p* < 0.0001) but showed no significant difference on CD4^+^ T cells between PD patients and controls (*p* > 0.05). Compared with healthy controls, PD patients had significantly increased peripheral blood C‐reactive protein (CRP) levels (*p* < 0.001) but remarkably decreased monocytes, lymphocytes, and MHR (*p* < 0.01). Collectively, the upregulated expression of CXCR3 and CXCR6 predominantly on CD8^+^ T lymphocytes may contribute to PD pathogenesis, though no significant correlation between the expression of these receptors and peripheral inflammation was observed.

## Graphical abstract

CXCR3 and CXCR6 expression may be dependent on CD8^+^ T lymphocytes to participate in the pathogenesis of PD, while has no effects on peripheral inflammation.

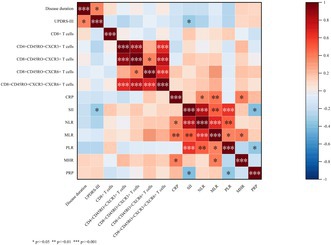

## 1 Introduction

Pathological features of Parkinson's disease (PD) include the degenerative loss of nigrostriatal dopaminergic neurons, the aggregation of α‐synuclein, and neuroinflammation [1]. In recent years, neuroinflammation has been recognised as a crucial factor in the pathogenesis of PD. Prolonged neuroinflammation disrupts the blood–brain barrier, thereby facilitating the entry of peripheral immune cells and chemokines into the central nervous system [2]. Moreover, the reciprocal activation of peripheral inflammation triggers the release of inflammatory cytokines and chemokines, ultimately contributing to neurodegeneration [3].

CXC chemokines and their receptors, as key mediators of chronic inflammation, have been extensively investigated regarding their specific mechanisms in neuroinflammation. Specifically, the CXCL16/CXCR6 axis exerts a protective effect against ischemic neuronal injury and regulates neurotransmitter release in the hippocampus by mediating glial cell‐neuron communication [4]. In contrast, the CXCL10‐CXCR3 signalling pathway induces microglial activation and abnormal neuroinflammation through mediating the infiltration of T cells into the brain [5]. As an important component of neuroinflammation, cellular immunity involving chemokines and their receptors continues to be studied. Notably, in PD, there is a complex interplay between peripheral inflammation and neuroinflammation; these two processes interact with each other and jointly drive disease progression. Emerging evidence suggests that components of the haematological inflammatory system, particularly neutrophils, infiltrate and accumulate in the perivascular space, which in turn leads to blood–brain barrier impairment and vascular diseases [6, 7]. The systemic immunoinflammatory index (SII) is regarded as a promising indicator of systemic inflammatory response, and it exhibits a close correlation with the development of cerebral small vessel diseases [8]. Moghaddam et al. validated the association between neutrophil‐to‐lymphocyte ratio (NLR) and striatal binding ratio in PD [9]. Additionally, NLR and platelet‐to‐lymphocyte ratio (PLR) have been utilised as potential biomarkers of peripheral inflammation to distinguish PD from progressive supranuclear paralysis (PSP) [10] and multiple system atrophy‐Parkinsonian (MSA‐P) [11]. Monocyte‐to‐high‐density lipoprotein ratio (MHR) holds potential as an indicator of early peripheral inflammation in PD and Parkinson plus syndrome (PPS) [12]. Furthermore, MHR, NLR, and red blood cell distribution width to platelet ratio (RPR) may serve as biomarkers to predict peripheral inflammation in the context of multiple system atrophy (MSA) [13]. Based on the aforementioned studies, it is imperative to select a panel of inflammatory markers to confirm peripheral inflammation in patients with PD.

In the present study, peripheral venous blood samples were collected from both PD patients and healthy controls to detect potential differences of circulating CXCR6^+^ T cells and CXCR3^+^ T cells, and to explore whether CXCR6 and CXCR3 could serve as potential biomarkers for PD. Furthermore, routine blood tests of two groups was obtained, and immune indices derived from SII, NLR, MLR, platelet‐to‐lymphocyte ratio (PLR), MHR and RPR were used to validate the feasibility of evaluating peripheral inflammation, thus providing a noval research basis for elucidating the pathogenesis of PD and identifying potential early diagnosis biomarkers.

## 2 Methods

### 2.1 Clinical Information

A total of 36 PD patients who were admitted to the Department of Neurology of the General Hospital of the Chinese People's Liberation Army from September 2024 to April 2025 and 26 healthy controls matched with the age and gender of the PD subjects were recruited. Clinical data including gender, age, disease duration, severity stage (UPDRS‐III), use of levodopa or immunomodulatory medications, BMI, smoking status, clinical history of hypertension, coronary artery disease, hyperlipidaemia, C‐reactive protein (CRP), cystatin C, total leukocyte count, monocytes, lymphocytes, and neutrophils, SII, NLR, MLR, PLR, MHR, and RPR were collected. The study design and methods were in accordance with the Declaration of Helsinki. All human experiments were conducted after written informed consent was obtained from all participants and the study was approved by the Institutional Review Board of the General Hospital of the Chinese People's Liberation Army (S2024‐719‐01).

#### 2.1.1 Inclusion Criteria

PD subjects fulfilled the internationally recognised clinical diagnostic criteria for PD from the UK Parkinson's Disease Brain Bank.

#### 2.1.2 Exclusion Criteria

The PD group excluded (1) those with other causes of PD syndrome, such as pharmacological causes, vascular causes, idiopathic tremor, encephalitis, a history of traumatic brain injury or surgery, and those with PD overlap syndrome; (2) those with a history of schizophrenia, Alzheimer's disease, atopic dermatitis, cerebrovascular accidents, diabetes mellitus, pituitary tumour, growth hormone overdose, malignancy, acute or chronic inflammatory infections, autoimmune disorders, severe cardiac, hepatic and renal failure; (3) those who have used glucocorticoid, growth hormone, growth inhibitor‐type or immunosuppressant drugs within the previous month; (4) those with other causes of limb dysfunction.

The healthy control group excluded (1) those who had infections or took antibiotics in the last 1 week; (2) those who had motor disorders, abnormal levels of RPR, or other novel inflammatory index values.

### 2.2 Main Reagents

Sterile PBS buffer (1× PBS, 500 mL, Biosharp), fetal bovine serum (500 mL, Biosharp), human lymphocyte separation medium (500 mL, Sigma‐Aldrich), RPMI 1640 medium (500 mL, Sigma‐Aldrich), red blood cell lysing buffer (100 mL, BD Biosciences), PerCP/Cyanine5.5 anti‐human CD3 antibody (OKT3, BioLegend), FITC anti‐human CD4 antibody (RPA‐T4, BioLegend), Brilliant Violet 510 anti‐human CD8 antibody (SK1, BioLegend), APC anti‐human CD45RO antibody (UCHL1, BioLegend), Brilliant Violet 421 anti‐human CD183 (CXCR3) antibody (G025H7, BioLegend), PE anti‐human CD186 (CXCR6) antibody (K041E5, BioLegend).

### 2.3 Blood Collection and PBMC Isolation

Briefly, 5 mL of venous blood was collected from the antecubital vein of each participant, placed in an EDTA‐coated vial, mixed with an equal volume of ice‐cold PBS, and the mixture was centrifuged at 400 × g for 30 min at 22°C to separate the plasma and white blood cell layers. White blood cells were collected and placed in a mixture of 5 mL of fetal bovine serum and RPMI‐1640 medium (the ratio of the two is 1:9), and centrifuged at 300 × g for 10 min at 22°C; the supernatant was discarded. The above operation was repeated to complete the washing three times, the supernatant was discarded, and the cells were suspended in an appropriate amount of RPMI‐1640 medium, and the density and viability of the cells were determined by observation under a microscope using the trypan blue method. All peripheral blood mononuclear cell (PBMC) samples were isolated via Ficoll density gradient centrifugation within 6 h of collection, followed immediately by surface staining and flow cytometry analysis.

### 2.4 Flow Cytometry Analysis

Cells were stained with fluorescent dye‐coupled antibodies for 15 min at 4°C, followed by an erythrocyte lysis procedure, washed with FACS buffer (2% FCS in PBS), and then resuspended in FACS buffer and fixed with PFA before collection. Samples were collected on a Canto II cytometer (BD Biosciences), and data were analysed using the FlowJo software.

### 2.5 Statistical Analysis

The results were analysed using FlowJo 10.5.3 software. Different subgroups of each indicator were analysed using SPSS 26.0 software. GraphPad Prism 9.5 software and Origin 2024 software were used for statistical analysis and graphing of data. Descriptive information was expressed as ratios, proportions and mean ± standard deviation (SD), and the Kolmogorov–Smirnov test was used to detect whether the data met the normal distribution. If it met the normal distribution, the unpaired *t*‐test was used for comparison between groups, while if the data were skewed, the non‐parametric Mann–Whitney *U* test was used for comparison between groups. *p* < 0.05 indicated that the differences were of statistical significance. A post hoc power analysis was carried out using G*Power software (version 3.1.9.2) for both primary and secondary outcomes, with a significance level of *α* = 0.05 and based on the study's actual sample size.

## 3 Results

### 3.1 Comparison of General Information

A total of 36 PD cases were included (27 males and 9 females; mean age 68.50 ± 11.50 years). The median disease duration was 2.00 years [interquartile range (IQR) 0.81–3.00], and the mean UPDRS‐III motor score was 16.17 ± 1.27. At baseline, 20 patients were receiving levodopa or immunomodulatory medications, while 16 were not. There were 26 cases in the control group 19 males and 7 females; mean age 68.81 ± 9.19 years. Compared with the healthy control group, there were no statistically significant difference in total leukocytes and neutrophils counts in the PD group, which further excludes infections and other interfering factors. The differences in C‐reactive protein, cystatin C, monocytes, lymphocytes, and MHR were statistically significant compared with those in the healthy control group (*p* < 0.05). There was no statistically significant difference in smoking, BMI, hypertension, diabetes mellitus, coronary heart disease, and hyperlipidaemia between the two groups (*p* > 0.05), which was summarised in Table 1. Compared with patients who did not use levodopa or immunosuppressants, the frequency of CD4^+^CD45RO^−^CXCR6^+^ T cells and CD4^+^CD45RO^−^CXCR3^+^CXCR6^+^ T cells in patients using levodopa or immunosuppressants were significantly decreased (*p* < 0.05), which was summarised in Table 2. Post hoc power analysis revealed that the statistical power for all inflammatory marker comparisons was below the generally accepted threshold of 80% (*α* = 0.05). Therefore, non‐statistically significant results herein should be considered preliminary findings, which require validation through larger‐scale studies.

**TABLE 1:** Clinical and demographic findings of subjects.

	PD	HC	*p* value
Sample size	36	26	
Male:Female	27:9	19:7	0.546^a^
Age in years (mean ± SD)	68.50 ± 11.50	68.81 ± 9.19	0.868^b^
Smoking((Y: N))	15:21	11:15	0.960^a^
BMI (mean ± SD)	25.92 ± 0.46	25.55 ± 0.72	0.652^b^
High blood pressure (Y: N)	20:16	17:9	0.600^a^
Diabetes (Y: N)	11:25	10:16	0.592^a^
Coronary heart disease (Y:N)	9:27	5:21	0.760^a^
Hyperlipidemia (Y: N)	18:18	9:17	0.301^a^
Disease duration	2.00 (0.81, 3.00)		
UPDRS‐III (mean ± SD)	16.17 ± 1.27		
Levodopa or immunomodulatory medications (Y: N)	20:16		
C‐reactive protein (mg/L)***	0.70 (0.42, 2.55)	0.10 (0.10, 0.21)	< 0.001^c^
Cystatin C (mg/L)*	0.98 (0.79, 1.16)	1.10 (1.01, 1.19)	0.031^c^
Total leucocyte count (×1000/μL)	5.27 (4.39, 6.84)	5.79 (5.08, 7.59)	0.164^c^
Monocyte count (×1000/μL)**	0.39 (0.32, 0.47)	0.50 (0.41, 0.60)	0.004^c^
Lymphocyte count (×1000/μL)**	1.33 (1.09, 1.72)	1.76 (1.32, 2.31)	0.008^c^
Neutrophil count (×1000/μL)	3.26 (2.65, 4.50)	3.51 (2.72, 4.32)	0.808^c^
SII	466.88 (335.41, 615.82)	400.21 (284.87, 569.49)	0.266^c^
NLR	2.62 (1.65, 3.22)	2.23 (1.54, 2.61)	0.091^c^
MLR	0.30 (0.21, 0.38)	0.28 (0.23, 0.34)	0.700^c^
PLR	137.66 (107.40, 167.92)	126.77 (89.49, 141.47)	0.101^c^
MHR**	0.33 (0.25, 0.48)	0.46 (0.33, 0.62)	0.004^c^
RPR	0.07 (0.06, 0.09)	0.06 (0.05, 0.08)	0.153^c^

*Note:* The data are presented as the numbers of patients (%), means ± SDs, or medians (Q1, Q3), unless indicated otherwise. *p* values are for comparisons of the two groups.

Abbreviations: CRP, C‐reactive protein; HC, Healthy control; MHR, Monocyte to high‐density lipoprotein ratio; MLR, Monocyte to lymphocyte ratio; N (No); NLR, Neutrophil to lymphocyte ratio; PD, Parkinson's disease; PLR, Platelet to lymphocyte ratio; Q1, quartile one; Q3, quartile three; RPR, Red cell distribution width (RDW) to platelet ratio; SD, standard deviation; SII, Systemic immune‐inflammation index; Y (Yes).

^a^ Chi‐square test.

^b^ Unpaired *t*‐test.

^c^ Mann–Whitney *U* test.

* *p* < 0.05.

** *p* < 0.01.

*** *p* < 0.001 vs. HC.

**TABLE 2:** Comparison of levodopa or immunomodulatory medication users versus non‐users.

Levodopa or immu‐nomodulatory medications	Users	Non‐users	t/Z‐value	*p* value
CD3^+^				
CD4^+^	41.29 ± 4.77	35.04 ± 4.07	0.968	0.340^a^
CD8^+^	33.01 ± 2.99	36.45 ± 3.52	−0.750	0.458^a^
CD4^+^CD45RO^+^				
CXCR3^+^	27.48 (16.41, 59.23)	15.68 (6.47, 45.39)	−1.305	0.192^b^
CXCR6^+^	1.86 (1.45, 2.90)	2.29 (1.39, 3.92)	0.159	0.874^b^
CXCR3^+^CXCR6^+^	0.99 (0.49, 1.51)	0.65 (0.36, 1.16)	−0.828	0.408^b^
CD4^+^CD45RO^−^				
CXCR3^+^	4.49 (2.99, 13.50)	5.10 (2.51, 7.63)	−0.541	0.588^b^
CXCR6^+^ **	0.45 (0.34, 0.73)	1.19 (0.77, 1.84)	3.152	0.002^b^
CXCR3^+^CXCR6^+^ *	0.11 (0.07, 0.25)	0.24 (0.15, 0.55)	2.072	0.038^b^
CD8^+^CD45RO^+^				
CXCR3^+^	37.90 (19.42, 54.06)	15.64 (4.90, 51.64)	−1.337	0.181^b^
CXCR6^+^	11.52 (6.55, 19.70)	13.49 (6.90, 25.92)	0.287	0.774^b^
CXCR3^+^CXCR6^+^	3.81 (1.97, 5.61)	2.40 (0.62, 6.32)	−0.955	0.340^b^
CD8^+^CD45RO^−^				
CXCR3^+^	28.27 (6.84, 42.54)	10.00 (3.81, 41.22)	−1.082	0.279^b^
CXCR6^+^	3.94 (2.42, 9.21)	5.95 (1.96, 9.74)	−0.350	0.726^b^
CXCR3^+^CXCR6^+^	0.88 (0.24, 2.03)	0.36 (0.17, 1.10)	−1.369	0.171^b^

*Note:* This figure illustrates the statistical differences in the proportion of each cell subpopulation between users of levodopa or immunomodulatory medications and non‐users. This figure presents statistical differences in the proportion of each cell subpopulation between users of levodopa or immunomodulatory medications and non‐users.

^a^ Unpaired *t*‐test.

^b^ Mann–Whitney *U* test.

* *p* < 0.05.

** *p* < 0.01.

*** *p* < 0.001 versus HC.

### 3.2 Flow Cytometry Assay

**FIGURE 1:**
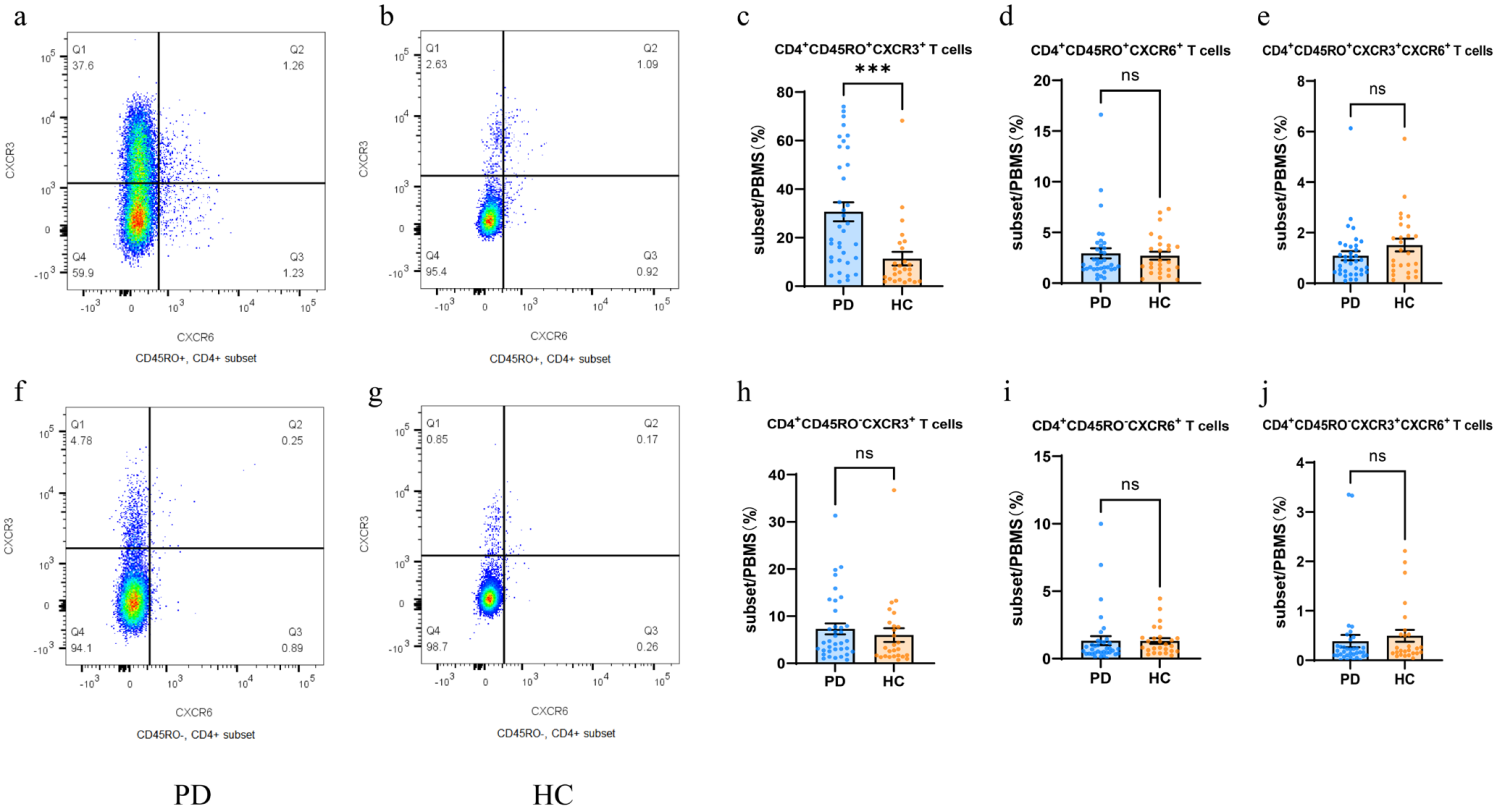
Flow cytometry plot of representative CD4^+^ lymphocytes. Peripheral blood was collected, stained with fluorescently labelled antibodies, subjected to erythrocyte lysis, then washed and fixed before analysis by flow cytometry. (a, b) CD4^+^CD45RO^+^ T lymphocytes were selected; (c–e) Expression of CXCR3 was significantly elevated on CD4^+^CD45RO^+^ T cells compared to controls; (f, g) CD4^+^CD45RO^−^ T lymphocytes were selected. (h–j) Expression of CXCR3 and CXCR6 in CD4^+^CD45RO^−^T cells did not differ between the two groups. Shown are representative flow cytometry images. Data are expressed as mean ± SEM (PD: *n* = 36, HC: *n* = 26). Statistical comparisons were performed using Mann–Whitney *U* test, * *p* < 0.05, **p < 0.01, ***p < 0.001, ****p < 0.0001 versus HC.

**FIGURE 2:**
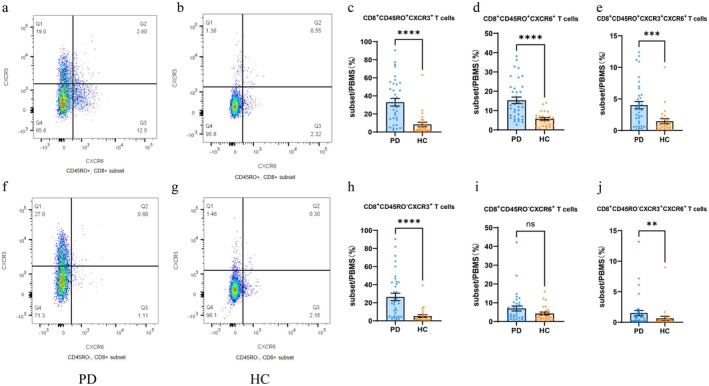
Flow cytometry plot of representative CD8^+^ lymphocytes. Peripheral blood was harvested, stained using fluorescently labelled antibodies, processed for erythrocyte lysis, then washed and fixed before being subjected to flow cytometry analysis. (a, b) CD8^+^CD45RO^+^ T lymphocytes were selected; (c–e) The expression of CXCR3 and CXCR6 on CD8^+^CD45RO^+^ T cells from PD patients was significantly higher than that in the control group; additionally, the dual expression of CXCR3 and CXCR6 in PD patients' CD8^+^CD45RO^+^ T cells was significantly elevated. (f, g) CD8^+^CD45RO^−^ T lymphocytes were selected. h–j: CXCR3 was significantly expressed on PD patients' CD8^+^CD45RO^−^ T cells. Shown are representative flow cytometry images. Data are expressed as mean ± SEM (PD: *n* = 36, HC: *n* = 26). Statistical comparisons were performed using Mann‐Whitney *U* test, *p < 0.05, **p < 0.01, ***p < 0.001, ****p < 0.0001 versus HC.

**FIGURE 3:**
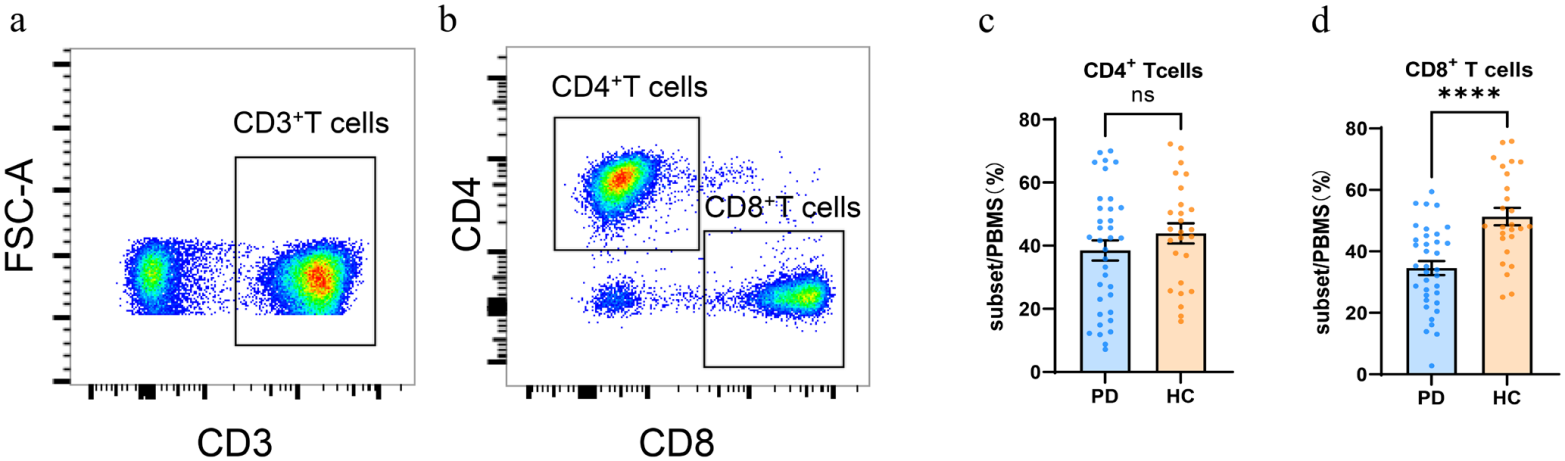
Strategies for chemokine receptor analysis in peripheral blood of PD patients (*n* = 36) versus HC (*n* = 26). (a, b) Lymphocytes were circled according to the forward scatter (FSC) and side scatter (SSC) in lymphocytes, while live CD3^+^ T cells were gated based on the expression of CD3, and then CD4^+^ T and CD8^+^ T cells were further gated. c and d: CD8^+^ T cells were significantly higher in the control group. Data are expressed as mean ± SEM. Statistical comparisons were performed using Mann–Whitney *U* test, *p < 0.05, **p < 0.01, ***p < 0.001, ****p < 0.0001 versus HC.

For the flow cytometry assay, lymphocytes were gated according to the forward scatter (FSC) and side scatter (SSC) in lymphocytes, while live CD3^+^ T cells were gated based on the expression of CD3. CD4^+^ T and CD8^+^ T cells were further gated, with CD45RO‐negative and CD45RO‐positive cell populations being sorted out from CD4^+^ T and CD8^+^ T populations, respectively. Cells were divided into four clusters: CXCR3^−^CXCR6^−^, CXCR3^+^CXCR6^−^, CXCR3^+^CXCR6^+^, and CXCR3^−^CXCR6^+^. Multi‐colour analysis was performed using FlowJo software, and the frequency of each T lymphocyte subpopulation was calculated. CD8^+^ T cells (Z = −4.660, *p* < 0.001) were significantly reduced in the PD group. Representative graphs of CD4^+^ T lymphocytes were shown in Figure 1, and representative graphs of CD8^+^ T lymphocytes were shown in Figure 2. Compared with the healthy control group, the PD group showed a higher frequency of CD4^+^CD45RO^+^CXCR3^+^ T cells (Z = −3.738, *p* < 0.001), CD8^+^CD45RO^+^CXCR3^+^ T cells (Z = −4.622, *p* < 0.001), CD8^+^CD45RO^+^CXCR6^+^ T cells (Z = −4.237, *p* < 0.001), CD8^+^CD45RO^+^CXCR3^+^CXCR6^+^ T cells (Z = −3.531, *p* < 0.001), CD8^+^CD45RO^−^CXCR3^+^ T cells (Z = −4.579, *p* < 0.001), and CD8^+^CD45RO^−^CXCR3^+^CXCR6^+^ T cells (Z = −2.940, *p* = 0.003). These results were shown in Table 3 and Figures 1, 2, 3.

**TABLE 3:** Comparison of T cell subset frequencies between PD group and healthy controls.

	Proportion (%) PD	HC	*t/Z*‐value	*p* value
CD3^+^				
CD4^+^	38.51 ± 3.21	43.89 ± 3.18	−1.157	0.252^a^
CD8^+^ ***	34.54 ± 2.27	51.52 ± 2.85	−4.660	< 0.001^a^
CD4^+^CD45RO^+^				
CXCR3^+^ ***	23.67 (10.86, 55.43)	6.93 (2.61, 16.15)	−3.738	< 0.001^b^
CXCR6^+^	1.86 (1.42, 3.65)	2.01 (1.23, 3.74)	−0.093	0.930^b^
CXCR3^+^CXCR6^+^	0.87 (0.44, 1.48)	1.25 (0.50, 2.27)	−0.221	0.134^b^
CD4^+^CD45RO^−^				
CXCR3^+^	4.55 (2.60, 10.35)	3.14 (1.51, 8.07)	−1.213	0.229^b^
CXCR6^+^	0.70 (0.40, 1.25)	0.99 (0.57, 1.56)	1.569	0.118^b^
CXCR3 + CXCR6^+^	0.17 (0.10, 0.29)	0.22 (0.14, 0.54)	1.485	0.138^b^
CD8^+^CD45RO^+^				
CXCR3^+^ ***	27.91 (9.17, 53.31)	3.20 (1.58, 11.06)	−4.622	< 0.001^b^
CXCR6^+^ ***	12.37 (6.90, 22.00)	5.70 ± 3.51	−4.237	< 0.001^b^
CXCR3^+^CXCR6^+^ ***	3.29 (0.80, 5.61)	0.90 (0.32, 1.81)	−3.531	< 0.001^b^
CD8^+^CD45RO^−^				
CXCR3^+^ ***	19.14 (4.48, 42.54)	1.72 (0.80, 6.63)	−4.579	< 0.001^b^
CXCR6^+^	4.61 (2.02, 9.35)	2.66 (1.51, 5.87)	−1.469	0.144^b^
CXCR3^+^CXCR6^+^ **	0.73 (0.21, 1.78)	0.19 (0.09, 0.46)	−2.940	0.003^b^

*Note:* This figure presents statistical differences in the proportion of each cell subpopulation between Parkinson's disease (PD) patients and healthy controls (HC).

^a^ Unpaired *t*‐test.

^b^ Mann–Whitney *U* test.

* *p* < 0.05.

** *p* < 0.01.

*** *p* < 0.001 versus HC.

### 3.3 Differential Expression Correlation Coefficient Plot

**FIGURE 4:**
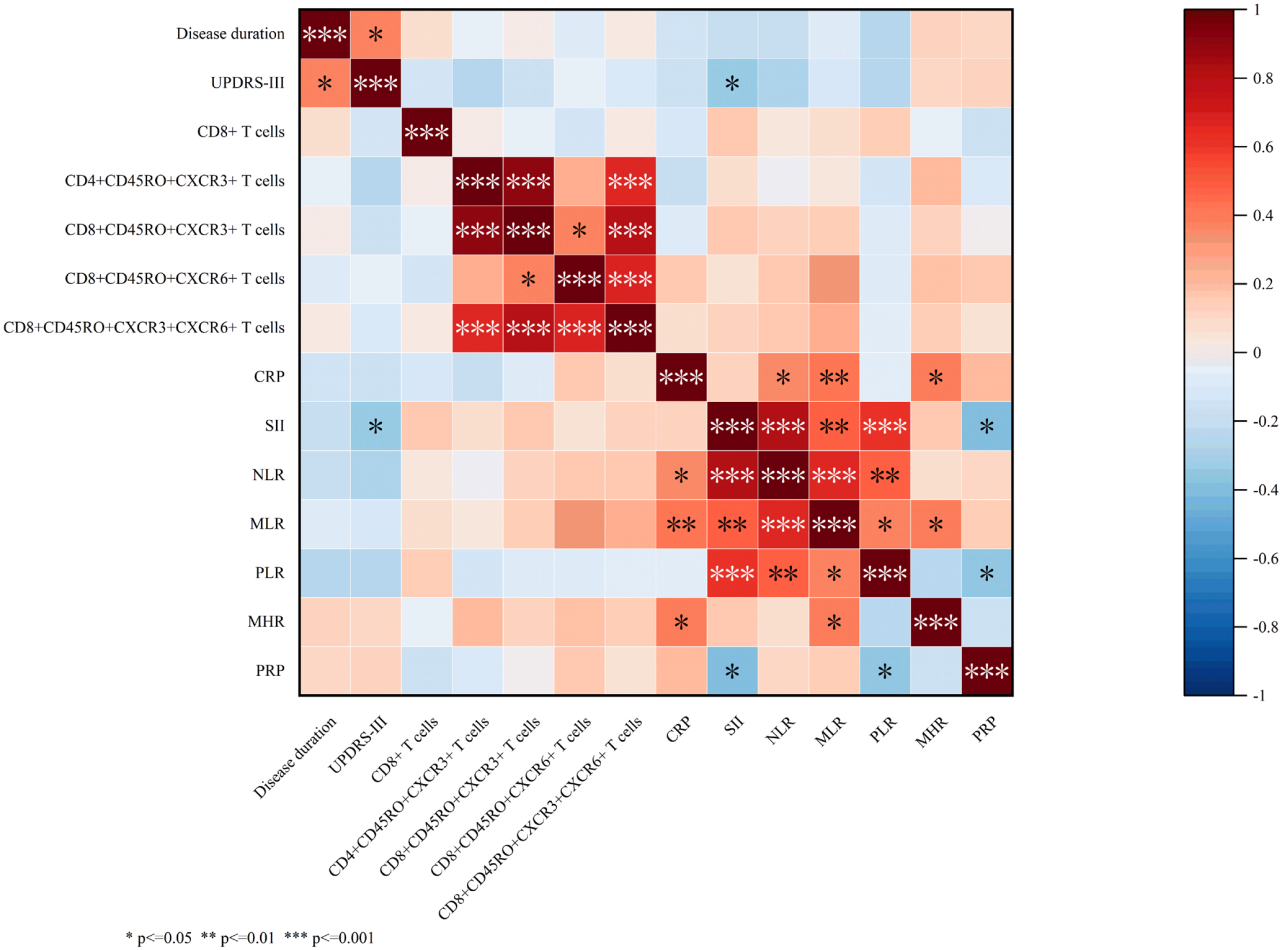
Heatmap of the correlation between CXCR3^+^/CXCR6^+^ T lymphocytes and Disease duration/UPDRS‐III/CRP/novel inflammatory markers (*Note:* Colour intensity represents the magnitude of Spearman's correlation coefficient; positive/negative correlations are indicated by different colour tones, as specified in the figure legend). Spearman's correlation analysis was used to assess correlations in both the Parkinson's disease (PD) group and healthy control (HC) group. *p < 0.05, **p < 0.01, ***p < 0.001; ns: No significance. CRP: C‐reactive protein, MHR: Monocyte to high‐density lipoprotein ratio, MLR: Monocyte to lymphocyte ratio, NLR: Neutrophil to lymphocyte ratio, PLR: Platelet to lymphocyte ratio, RPR: Red cell distribution width (RDW) to platelet ratio, SII: Systemic immune‐inflammation index.

**FIGURE 5:**
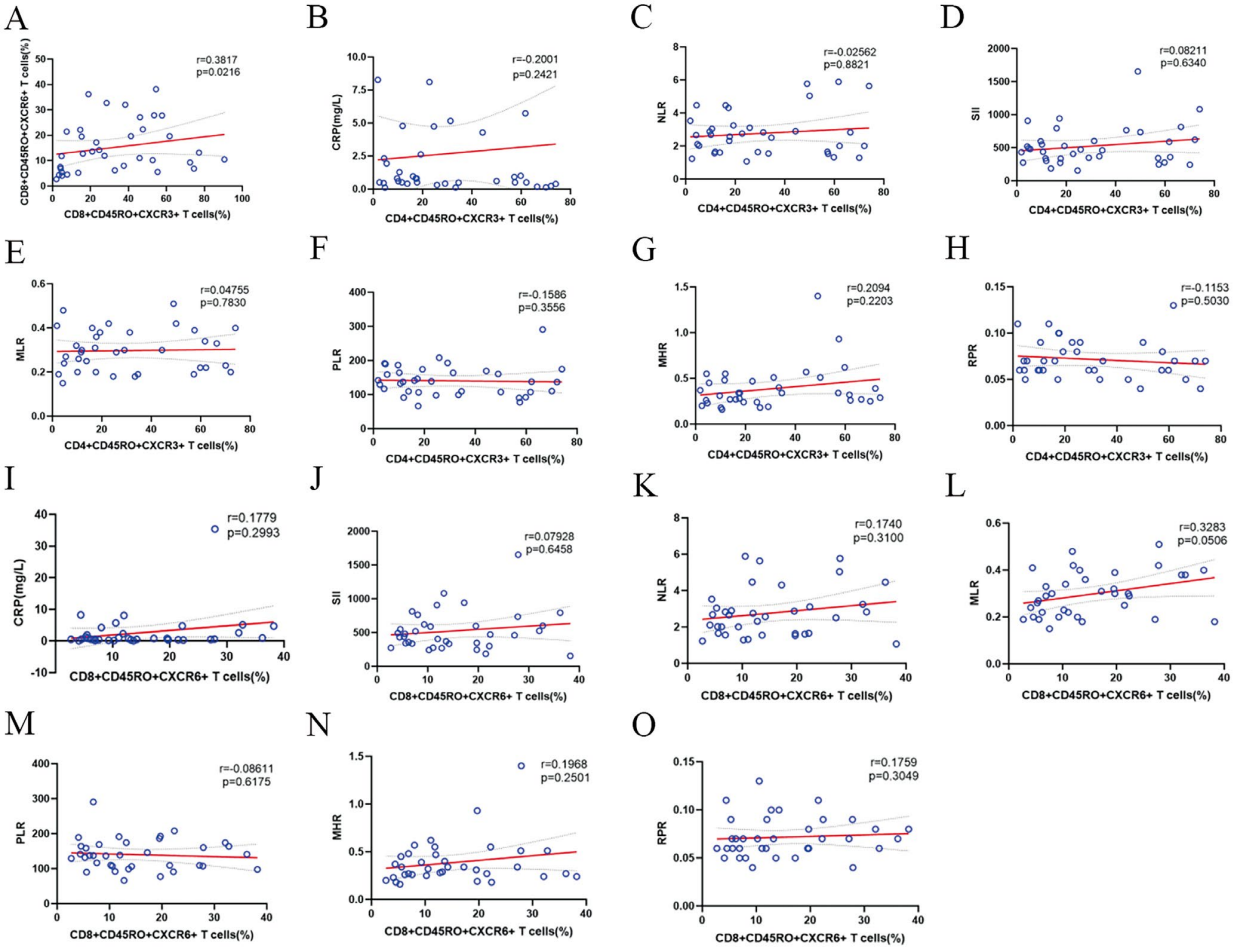
Correlation of CXCR3 and CXCR6 with CRP and novel inflammatory indicators in PD patients. (a) Correlation of CXCR3 and CXCR6 expressed on CTLs. (b–h). Correlation of CD4^+^ Effector T Cells expressing CXCR3 in the peripheral blood of PD patients with CRP and novel immune indicators. (i–o). Correlation of CTLs expressing CXCR6 in the peripheral blood of PD patients with CRP and novel immune indicators. *p < 0.05, **p < 0.01, ***p < 0.001, ns: No significance. CRP: C‐reactive protein, MHR: Monocyte to high‐density lipoprotein ratio, MLR: Monocyte to lymphocyte ratio, NLR: Neutrophil to lymphocyte ratio, PLR: Platelet to lymphocyte ratio, RPR: Red cell distribution width to platelet ratio, SII: Systemic immune‐inflammation index.

Correlation coefficient plots were produced for above indicators that were significantly different from the healthy control group (where *p* < 0.05). There were a total of 14 groups of indicators, including 5 groups of T‐cell indicators expressing chemokine receptors, 3 groups of clinical test indicators, and 6 groups of novel inflammation indicators. The correlation coefficient graph was used to analyse the correlation of disease duration, UPDRS‐III, and above different indicators. As shown in Figures 4 and 5, upon assessment, we found no evidence for a correlation between clinical features (including disease duration and UPDRS‐III scores) and peripheral abnormal cellular immunity in our cohort of PD patients. CXCR3‐expressing CD4^+^ effector T cells were highly correlated with cytotoxic T lymphocytes (CTLs, CD8^+^CD45RO^+^ T cells), and CXCR6‐expressing CTLs were only moderately correlated with CXCR3‐expressing CTLs.

## 4 Discussion

In this study, CD8^+^ T cells were significantly reduced in the PD group. CXCR3 was widely expressed on CD4^+^ effector T cells and CD8^+^ T cells in the peripheral blood of PD patients. CXCR6 in the peripheral blood was significantly expressed on CTLs in the PD group, while there was no statistically significant difference in CXCR6 expression on CD4^+^ T cells. There was widespread active peripheral inflammation in patients with PD. Novel inflammatory indicators calculated from monocytes and lymphocytes (which reflect peripheral inflammation) did not appear to correlate with T lymphocytes expressing the chemokines receptors CXCR3 and CXCR6.

Exposure to risk factors such as genetics, environment, and aging—which occur in the prodromal stage of PD and continues throughout the course of the disease—can initiate a central or peripheral inflammatory response that induces an abnormal immune response [14]. Cellular immunity plays a key role in the pathogenesis of PD. Chemokines and their receptors have been shown to be important in T cell activation, immune surveillance, and tolerance [15]. Nowadays, there is increasing interest in the mechanisms of action of chemokines and their receptors in neurodegenerative diseases as well as their role as biomarkers for disease diagnosis and severity. Eotaxins in the C‐C chemokines (CCL11, CCL24, and CCL26) and their receptor (CCR3) may serve as appropriate PD diagnostic or therapeutic targets [16]. The research related to C‐X‐C chemokines and their receptors in PD is still in its infancy.

Although the use of levodopa or immunomodulatory drugs affects the immune function, there was no statistically significant difference in the positive indicators of this study (such as CD8^+^CD45RO^+^CXCR6^+^ T cells) between users and non‐users. Therefore, the interference of the use of above‐mentioned drugs on the results of this study can be excluded. The chemokine receptor CXCR3 is expressed on CD4^+^ Th1 cells, naïve and memory CD8^+^ T cells, natural killer (NK) cells, and activated B cells [17]. In multiple sclerosis (MS) [18] and AD [19], activated T lymphocytes expressing CXCR3 could be recruited to sites of inflammation in the brain. Based on flow cytometry results in this study, CXCR3 was widely expressed on CD4^+^ effector T cells and CD8^+^ T cells in the peripheral blood of PD patients, and CD4^+^ T cells expressing CXCR3 can accumulate and produce interferon at Th1‐type inflammatory sites [20], which amplifies microglia‐mediated local inflammatory responses and thus exerts cytotoxic effects on dopaminergic neurons [21]. CXCR6 [22]—whose ligand is CXC chemokine ligand 16 (CXCL16)—was discussed by Piehl et al. [23], who emphasised the CXCL16‐CXCR6 signalling pathway as a potential mechanism of T‐cell entry into the AD brain. Su et al. [24] confirmed that CXCR6 exerted a protective effect on neurons in AD patients. Hou [25] found that CD4^+^CXCR6^+^ T cells secrete a variety of inflammatory factors and have a pathogenic effect on the experimental autoimmune encephalomyelitis (EAE) model. In our study, CXCR6 in the peripheral blood was significantly expressed on CTLs in the PD group, while there was no difference on CD4^+^ T cells. It is hypothesized that CXCR6 may be involved in peripheral‐central immune‐cell communications in PD sharing a neuroprotective mechanism similar to that in AD. Correlation analysis of CXCR3 and CXCR6 expressed on CTLs showed a significant positive correlation between CXCR3 and CXCR6 (*p* < 0.01) and a significant increase in CD8^+^CXCR3^+^CXCR6^+^ T cells compared to the healthy controls, suggesting that CXCR3 and CXCR6 may be dependent on CD8^+^ T lymphocytes to participate in the pathogenic process of PD. It is less likely that they play a pathogenic role by secreting inflammatory factors through CD4^+^ T cells. In the present study, peripheral CD8^+^ T lymphocytes were reduced in PD patients, with the possibility of infiltrating centrally to participate in aberrant cellular immunity.

Neutrophils, lymphocytes, and monocytes are present in the peripheral blood, and these types of cells play an important role in mediating inflammatory responses. Indicators of inflammation—a group of non‐specific parameters widely used to reflect the level of inflammation—are feasible for clinical use as a tool for routine assessment of the intensity of peripheral inflammation. Calculation of inflammatory indicators based on collected clinical data can lead to results consistent with other studies. Plasma CRP levels were significantly higher in PD patients than in the healthy control group, with circulating monocytes and lymphocytes being significantly lower [26] and MHR being lower than that in the healthy control group. There was widespread active peripheral inflammation in patients with PD, despite the fact that the mean values of NLR and PLR were higher than those of the healthy control group. This difference was not statistically significant, which is not in accordance with the findings of Akıl E [27] and Madetko [8]. However, in agreement with the results of Inci [7] and Ataç [28], these observed differences may be attributable to differences in disease duration and clinical severity.

In our study, no correlation was observed between the course of disease, disease severity, and abnormal peripheral cellular immunity in PD. CRP, a sensitive indicator of inflammatory activity, was significantly higher in the peripheral blood of PD than in the normal population, while it did not correlate with the expression of either CXCR3 or CXCR6, suggesting that peripheral inflammatory activity in PD is not associated with aberrant cellular immunity. The relationship between inflammatory indicators and the expression of CXCR3 and CXCR6 further validates the above conjecture, although monocytes are linked to CXCR3 through the expression of chemokines CXCL9 and CXCL10, and lymphocytes may respond to aberrant cellular immunity. Novel inflammatory indices calculated from monocytes and lymphocytes did not correlate with T‐lymphocytes expressing chemokines receptors CXCR3 and CXCR6. Thus, in the aberrant cellular immunity of PD, T lymphocytes expressing chemokine receptors CXCR3 and CXCR6 are involved in the pathogenesis of PD via CD8^+^ T lymphocytes; however, no correlation between their expression and peripheral inflammation has been observed. However, it has been shown that inflammatory cells can be infiltrated and accumulated in the perivascular space, leading to the disruption of the blood–brain barrier and enlargement of the perivascular space, exacerbating the inflammatory response and tissue damage in a vicious circle [29]. With the expansion of the study population, this novel inflammatory indicator (NLR) shows a trend of correlation with T lymphocytes expressing chemokine receptors CXCR3 and CXCR6, which may be due to the fact that peripheral inflammation further damages the blood–brain barrier and promotes the central migration of Tlymphocytes. According to our study, peripheral inflammation coexists with aberrant cellular immunity in PD patients, and chemokine receptors CXCR3 and CXCR6 can be involved in PD progression, which provides a new direction for the study of aberrant immune activation and neuroinflammation in PD.

A notable limitation of the present study is the relatively small sample size, which led to insufficient statistical power for several key analyses—particularly the comparisons of inflammatory markers. Post hoc power analysis confirmed that the statistical power for these comparisons was below the widely accepted 80% threshold (*α* = 0.05). This inadequacy may have compromised our ability to detect potential true differences between groups, and thus non‐significant findings should be interpreted with caution.

## 5 Conclusion

In conclusion, this study reveals that CXCR3^+^/CXCR6^+^ T lymphocytes may be involved in the pathogenesis of PD through a CD8^+^ T cell‐dependent pathway. Additionally, peripheral inflammation and aberrant cellular immunity in PD patients exist as independent processes. These findings provide a novel direction for investigating the immunopathological mechanisms of PD and screening potential targets for immunological intervention.

## Author Contributions

Yufeng Zhang, Zhuangzhuang Ren and Shuangshuang Jia: study design, data acquisition, interpretations of results, manuscript preparation and revisions. These authors have equal contributions to this study. Jinming Han, Xiaoling Zhong, Bo Fu, Haoran Wang and Shiping Xu: data acquisition, manuscript revision. Tingting Li and Feng Qiu: study design, manuscript revision. These authors are joint corresponding authors. All authors have read and agreed to the published version of the manuscript.

## Funding

The authors have nothing to report.

## Ethics Statement

The study design and methods were in accordance with the Declaration of Helsinki. All human studies were conducted after informed consent was obtained and approved by the Institutional Review Board of the General Hospital of the Chinese People's Liberation Army (S2024‐719‐01).

## Conflicts of Interest

The authors declare no conflicts of interest.

## Data Availability

The data that support the findings of this study are available on request from the corresponding author. The data are not publicly available due to privacy or ethical restrictions.
